# Supplementary material for: Higher body mass index is linked to altered hypothalamic microstructure
Source: Sci Rep. 2019 Nov 22;9:17373. doi: 10.1038/s41598-019-53578-4 (PMC6874651; doi:10.1038/s41598-019-53578-4)
Supplement: Supplementary file 1 — Supplementary Information [file 41598_2019_53578_MOESM1_ESM.docx]

**Supplementary information**

Higher body mass index is linked to altered hypothalamic microstructure

*K. Thomas, F. Beyer, G. Lewe, R. Zhang, S. Schindler, P. Schönknecht ,*

*M. Stumvoll, A. Villringer, A.V. Witte*

**Details about the multi-atlas fusion segmentation**

The algorithm included three main steps: First, the atlas images were non-linearly coregistered to the study-specific template using antsRegistration. The registration included a rigid-body transform, an affine transform and the non-linear ‘SyN’ registration step with four resolution levels. For exact settings of the parameters, see publicly available scripts. With the same command, the target image was non-linearly registered to the study-specific template. In a third step, an additional quick registration between the atlas images and the target image in the template space was performed. The quick registration used the same parameters as the full registration, but it excluded the fourth resolution level (**Fig. 4**).

All transforms were concatenated and applied in a single registration step to the hypothalami of the atlas images using antsApplyTransforms. This step yielded multiple labels of the hypothalamus in the target native space.

To fuse these labels, we applied STEPS (Similarity and Truth Estimation for Propagated Segmentations) implemented in NiftySeg (https://github.com/KCL-BMEIS/NiftySegSTEPS) which generated one multi-atlas based hypothalamic segmentation per target image.

| **Supplementary Table 1:** Measures of intra-rater, inter-rater reliability and spatial overlap for left and right hypothalamus according to semi-automated segmentation (n = 20) | | | | |
| --- | --- | --- | --- | --- |
|  | Left Hypothalamus | | Right Hypothalamus | |
|  | ICC^a^ | DSC | ICC^a^ | DSC |
| Rater |  |  |  |  |
| Rater 1 | .73 | .93 | .82 | .94 |
| Rater 2 | .95 | .96 | .89 | .96 |
| Rater 1 – Rater 2 | .83 | .88 | .88 | .89 |
| *ICC: Intra-class correlation coefficient, DSC: Dice similarity coefficient*  *^a^ ICC (1,1) was used to assess agreement within each rater, whereas ICC (3,1) was used to assess agreement between both raters* | | | | |

| **Supplementary Table 2:** Inter-rater reliability and percentage of overlap for left and right hypothalamic volume between the semi-automated segmentation sample and the two different multi-label fusion segmentation samples. | | | | |
| --- | --- | --- | --- | --- |
|  | Left Hypothalamus | | Right Hypothalamus | |
|  | ICC^a^ | DSC | ICC^a^ | DSC |
| Rater |  |  |  |  |
| Validation 1 (atlas) (n = 44) | .62 | .85 | .55 | .86 |
| Validation 2 (n = 24) | .67 | .85 | .73 | .85 |
| *ICC: Intra-class correlation coefficient, DSC: Dice similarity coefficient*  *^a^ ICC (3,1) was used to assess agreement between both approaches* | | | | |

| **Supplementary Table 3:** Inter-rater reliability and percentage of overlap for left and right hypothalamic MD between the semi-automated segmentation sample and the two different multi-label fusion segmentation samples. | | |
| --- | --- | --- |
|  | Left Hypothalamus | Right Hypothalamus |
|  | ICC^a^ | ICC^a^ |
| Rater | | |
| Validation 1 (atlas) (n = 44) | 0.97 | 0.98 |
| Validation 2 (n = 24) | 0.87 | 0.97 |
| *ICC: Intra-class correlation coefficient, DSC: Dice similarity coefficient*  *^a^ ICC (3,1) was used to assess agreement between both approaches* | | |

| **Supplementary Table 4:** Group characteristics of the samples used for multi-atlas fusion segmentation. Data is given as mean ± standard deviation and range (minimum – maximum). | | | | |
| --- | --- | --- | --- | --- |
|  | Semi-automated  n = 338 | Study-specific template  n = 150 | Validation 1 (atlas)  n = 44 | Validation 2  n = 24 |
| Age (years) | 55.0 ± 12.4  (21 – 78) | 55.1 ± 12.4  (21 – 78) | 56.3 ± 10.8  (34 – 76) | 59.2 ± 10.9  (40 – 78) |
| Sex (males/females) | 176/162 | 76/74 | 24/20 | 15/9 |
| Rater (1/2/both) | 166/152/20 | 73/69/8 | 21/19/4 | 11/9/4 |
| BMI (kg/m^2^) | 26.4 ± 3.9  (18 – 43) | 26.6 ± 3.8  (18-37) | 26.8 ± 3.8  (20 – 37) | 25.9 ± 2.8  (20 – 30) |
| *BMI: body mass index* |  |  |  |  |
